# Inferential Statistics from Black Hispanic Breast Cancer Survival Data

**DOI:** 10.1155/2014/604581

**Published:** 2014-02-11

**Authors:** Hafiz M. R. Khan, Anshul Saxena, Elizabeth Ross, Venkataraghavan Ramamoorthy, Diana Sheehan

**Affiliations:** ^1^Department of Biostatistics, Robert Stempel College of Public Health & Social Work, Florida International University, Miami, FL 33199, USA; ^2^Department of Health Promotion & Disease Prevention, Robert Stempel College of Public Health & Social Work, Florida International University, Miami, FL 33199, USA; ^3^Behavioral Science Research, 2121 Ponce De Leon, Coral Gables, FL 33134, USA; ^4^Department of Dietetics and Nutrition, Robert Stempel College of Public Health & Social Work, Florida International University, Miami, FL 33199, USA; ^5^Department of Epidemiology, Robert Stempel College of Public Health & Social Work, Florida International University, Miami, FL 33199, USA

## Abstract

In this paper we test the statistical probability models for breast cancer survival data for race and ethnicity. Data was collected from breast cancer patients diagnosed in United States during the years 1973–2009. We selected a stratified random sample of Black Hispanic female patients from the Surveillance Epidemiology and End Results (SEER) database to derive the statistical probability models. We used three common model building criteria which include Akaike Information Criteria (AIC), Bayesian Information Criteria (BIC), and Deviance Information Criteria (DIC) to measure the goodness of fit tests and it was found that Black Hispanic female patients survival data better fit the exponentiated exponential probability model. A novel Bayesian method was used to derive the posterior density function for the model parameters as well as to derive the predictive inference for future response. We specifically focused on Black Hispanic race. Markov Chain Monte Carlo (MCMC) method was used for obtaining the summary results of posterior parameters. Additionally, we reported predictive intervals for future survival times. These findings would be of great significance in treatment planning and healthcare resource allocation.

## 1. Introduction

### 1.1. Problem Statement: Breast Cancer in Black Hispanic Women

Cancer is defined as a process that induces irreversible mutations in cellular genetic processes resulting in uncontrolled growth and proliferations [1]. Tumor is defined as any abnormal growth of cancer cells that form a lump or mass. Human breast is primarily composed of fat, connective tissues, lymphatic vessels, and organized lobules of milk secreting glands. These lobules are connected exteriorly to the nipple via secretory ducts. Most breast cancers are carcinoma in situ (CIS) because they are confined by epithelial boundaries either to the duct (ductal carcinoma in situ (DCIS)) or to the lobule (lobular carcinoma in situ (LCIS)).

Breast cancer is one of the most common life-threatening cancers in women of any age group. According to the World Health Organization (WHO) report 2004, breast cancer comprises approximately 16% of all cancer types and causes 519,000 deaths annually worldwide. Surprisingly, 69% of these deaths occurred in developing countries, refuting the misconception that breast cancer is a disease of developed world [2]. According to the American Institute of Cancer Research (AICR), over 226,000 cases of breast cancer are diagnosed every year in USA and approximately 40,000 American women die of breast cancer every year [3].

### 1.2. Breast Cancer according to Race and Ethnicity

In the United States breast cancer is one of the most frequently diagnosed cancers across different racial and ethnic groups [2]. Race/ethnicity specific incidence rates remained fairly constant for all racial and ethnic groups during the years 2004–2008. [1]. Previously, it was believed that family history, socioeconomic status, levels of education, frequency of mammograms, and access to health care resources were some of the major determinants affecting the prognosis of the disease. However, recent studies have shown that racial and ethnic factors also contribute significant risk for the prognosis of the disease.

The American Cancer Society has found evidence that there are notable differences in breast cancer death rates between different states across various socioeconomic strata and between different racial/ethnic groups [1]. Although age is the strongest predictor for breast cancer risk, race/ethnicity could also be a major risk factor [1]. Since the early 1990s, breast cancer mortality rates have decreased among all ethnic groups except the American Indians/Alaska Natives, thereby showing another racial disparity associated with the disease. In the United States, White women are more likely to develop breast cancer than African-American, Hispanic, Asian, or American Indian/Alaska native women [1].

### 1.3. Hispanic Black Women

Although Hispanics are the fastest growing minority population in the United States, there are not much breast cancer statistics on Black Hispanics, specifically. Breast cancer data for Hispanics are usually tabulated under one ethnic group (Hispanic), therefore, race-specific breast cancer data for the Hispanic population is not readily available. Overall, the incidence and mortality rates of breast cancer among Hispanic (Black and White) women are lower than non-Hispanic White women [3]. Hispanic women (Black and White) show lower levels of awareness about the risk factors associated with the disease and have less access to health care facilities when compared to women of any other ethnicity.

Unfortunately, there are not many studies that elucidate breast cancer disparities among different races within the Hispanic ethnicity. Usual research findings describe breast cancer incidence, mortality, and death rates and other vital statistics associated with the disease among Hispanic women without any details about interracial differences within the ethnicity. Banegas and Li [4] have asserted that further study of specific breast cancer outcomes among the different races of Hispanic women could greatly enhance knowledge about the distribution and determinants of the disease in this high risk ethnic group. Their study showed that non-Hispanic Blacks had a 1.5–2.5-fold greater risk of having stage IV breast cancer types and 10–50% greater risk of breast cancer specific mortality compared to non-Hispanic whites [4]. This finding again shows the need for a study that tries to understand the current state of affairs about breast cancer survival in this subpopulation within United States.

### 1.4. Statistical Probability Models

Healthcare personnel has collected vast amounts of phenomic and genomic data which should be maximally utilized for research perspectives. These large databases should be tested with newer statistical methods and statistical probability models. This would be very useful to predict future patterns of disease morbidity and mortality, thereby enhancing our understanding the severity and outcomes.

Data may follow several statistical probability models like exponential, gamma, Weibull, normal, half-normal, log-normal, Rayleigh, inverse Gaussian, exponentiated exponential (EE), exponentiated Weibull (EW), beta generalized exponential (BGE), beta inverse Weibull (BIW), and so forth. Statistical models are immensely useful to characterize the data and derive reliable scientific inferences.

The biomedical and engineering fields often use exponentiated exponential model (EEM) for data modeling. The EEM, a generalization of exponential distribution, was introduced by Gupta and Kundu [5] and received rapid and widespread acceptance. The EEM considers two parameters: “shape” and “scale.” Moreover, Gupta and Kundu [6] observed that the EEM is similar to the Weibull family and suggested the possibility of using the EE distribution as a substitute for Weibull model.

A random variable *x* is said to follow the exponentiated exponential distribution if its probability density function (pdf) is given by

(1) p(x)=αλexp⁡{−(λx)}(1−exp⁡{−(λx)})α−1,

where *α* > 0 is the shape parameter and *λ* > 0 is the scale parameter. Gupta and Kundu [5] introduced the above mentioned density function for exponentiated exponential distribution. The random variable can be expressed as *x* ~ EE(*α*, *λ*). Some interesting applications of EEM include designing rainfall estimation in the Coast of Chiapas [7], analysis of Los Angeles rainfall data [8], and software reliability growth models for vital quality metrics [9]. A cure rate model based on the generalized exponential distribution that incorporates the effects of risk factors or covariates for the probability of an individual being a long-time survivor was proposed by Kannan et al. [10]. Also, Gompert'z form of exponentiated exponential model was used to predict squid axon voltage clamp conductance [11].

For a beta generalized exponential model, the probability density function is given by

(2) p(x)=αλB(a,b)exp⁡{−(λx)} ×(1−exp⁡{−(λx)})aα−1(1−(1−exp⁡{−(λx)})α)b−1,

where *α* > 0 is the shape parameter and *λ* > 0 is the scale parameter. There are two additional parameters, *a* > 0 and *b* > 0. The role of these parameters is to describe skewness and tail weight [12]. The BGE model generalizes some well-known models, for example, beta exponential and generalized exponential models, as special cases.

A Swedish physicist called Weibull [13] introduced the Weibull distribution primarily for examining the breaking strength of materials. The first EW model with bathtub shaped distribution and unimodal failure rates was introduced by Mudholkar and Srivastava [14]. Since then, application of the EW model to analyze lifetime data has been recommended by Nassar and Eissa [15] and Choudhury [16].

For the EW model, the probability density function (pdf) is given by

(3) p(x)=αβλxβ−1exp⁡{−(λxβ)}(1−exp⁡{−(λxβ)})α−1,

where *α* > 0 and *β* > 0 are the shape parameters and *λ* > 0 is the scale parameter.

The BIW model has several applications for problems in engineering, health, and medical fields. It shows best fit for several data sets, for instance, the amount of time taken for breakdown of insulating fluids subjected to tensions [17]. For the BIW model, the probability density function (pdf) is given by

(4) p(x)=βx−(β+1)B(a,b)exp⁡{−(x−β)}(exp⁡{−(x−β)})α−1 ×(1−exp⁡{−(x−β)})b−1,

where *β* represents the shape parameter and two parameters, *a* > 0 and *b* > 0, represent skewness and tail weight.

A novel Bayesian method can be used to derive the posterior probability for the parameters to calculate posterior inference. Model parameters and data are considered random variables in a Bayesian estimation technique. Their joint probability distribution is stated by a probabilistic model. Data are considered as “observed variables” and parameters as “unobserved variables” in a Bayesian method. Multiplying likelihood and prior gives the joint distribution for the parameters. The “prior” contains information about the parameter. The likelihood depends on the model of underlying process and measured as a conditional distribution which specifies the probability of the observed data. All the information available about the parameters is combined by prior and likelihood. By manipulating the joint distribution of prior and likelihood, inference about parameters of the probability model can be derived from the given data. The Bayesian inference intends to develop the posterior distribution of the parameters for given sets of observed data.

Readers can refer to Berger [18], Geisser [19], Bernardo and Smith [20], Ahsanullah and Ahmed [21], Gelman [22], and Baklizi [23, 24] for further information on Bayesian methods. Khan et al. [25], Thabane [26], Thabane and Haq [27], Ali-Mousa and Al-Sagheer [28], and Raqab [29, 30] have discussed several additional applications of Bayesian method for predictive inferences.

Objectives of this paper include (i) studying some demographic and socioeconomic variables; (ii) reviewing right skewed models EE, EW, BGE, and BIW; (iii) justifying that the given sample data follows a specific model by applying model selection criteria through goodness of fit tests; (iv) performing a Bayesian analysis of the posterior distribution of the parameters; and (v) deriving Bayesian predictive model for future response.

The structural organization of this paper is as follows: Section 2 includes a real example of breast cancer data discussed in detail; Section 3 includes the measure of goodness of fit tests, log-likelihood functions, and the posterior inference for the model parameters for race/ethnicity (Black Hispanic females only); Section 4 includes the Bayesian predictive model which includes the likelihood function, posterior density function, and the predictive density for a future response given a set of observations from the best model; Section 5 includes the results and discussions; and Section 6 includes the conclusion.

## 2. Real Life Data Example

Breast cancer data (*N* = 657,712) from Surveillance, Epidemiology, and End Results (SEER, 1973–2009) website has been used as a real life data example. Data on breast cancer patients collected from twelve states have been stored in SEER database. Stratified random sampling scheme was used to pick nine sates randomly from these twelve states. Data from these nine states included state-wise race/ethnicity categories for breast cancer distribution.

This data included 4,269 males and 653,443 females. Since breast cancers are rare in males, data from females only were used in our analysis. A simple random sampling (SRS) method was used to select 298 female subjects from Black Hispanic data.

**Figure 1:**
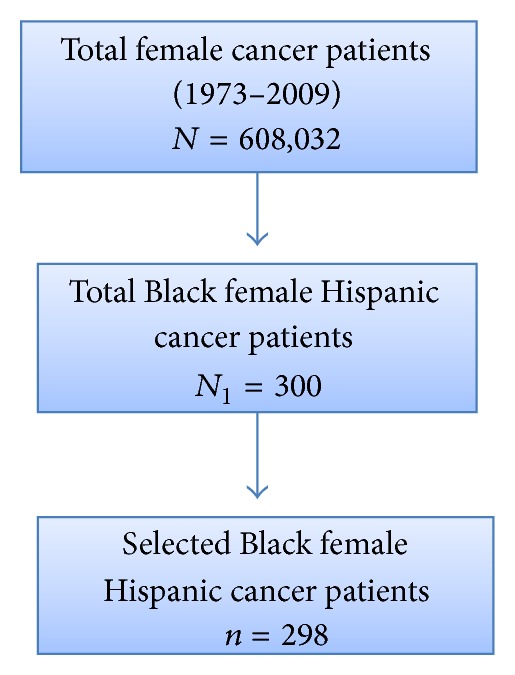
Selection of stratified random sample of Black Hispanic breast cancer patients from SEER (1973–2009) dataset represented as a pedigree chart.

**Figure 2:**
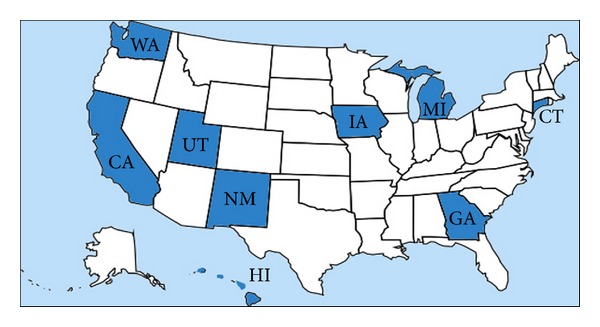
The nine states from which the Black Hispanic breast cancer patients were selected. *Note*. The Black Hispanic females (*n* = 298) patients were randomly selected from the dark blue colored nine states in Figure 2.

Figure 1 shows the pedigree chart for the selection of Black Hispanic breast cancer patients out of total female breast cancer patients *N* = 608,032. In the total population, there were 300 Black Hispanic female patients, but data were missing for 2 participants.  Figure 2 describes the nine states (dark blue regions), which were randomly selected and were followed by a random selection of Black Hispanic breast cancer patients.

The descriptive statistics (frequency distribution and summary statistics) are shown in Tables 1 and 2, respectively.  Table 1 contains the state wise frequency and its corresponding percentages for the selected patients.  Table 2 has the descriptive statistics (mean, standard deviation, median, quartiles, and variance) for some demographic characteristics (age at diagnosis, survival times, and marital status at diagnosis) of the selected random sample of Black Hispanic breast cancer patients.

We selected 2,000 non-Hispanic Blacks out of 53,531 Black non-Hispanics for comparing with 298 Black Hispanics in this sample. The mean survival for non-Hispanic Blacks was 66.76 (standard deviation 30.20) and for Black Hispanics 71.38 (standard deviation 61.33). Cox Proportional Regression was used to calculate hazard ratios by ethnicity. Hazard ratios compare the probability of an event occurring in one group versus another and take into account the time elapsed until the event should occur. In survival analysis, the event under consideration is death. A hazard ratio of 1.0 represents an equal risk of death between the groups being compared, a hazard ratio above 1.0 means an increased risk of death, and a hazard ratio below 1.0 represents a decreased risk of death compared to the referent group. In this analysis, we used non-Hispanic Blacks as a referent group. Statistical significance is established if the 95% confidence interval did not include 1. Non-Hispanic Blacks had a significantly increased risk of death compared to Black Hispanics (Hazard ratio: 1.445 95% Wald Robust Confidence Limits 1.210–1.724; 95% Profile Likelihood Confidence Limits 1.265–1.659). When compared to non-Hispanic Blacks, Hispanic Blacks had a significantly decreased risk of death (hazard ratio: 0.692 95% Wald Robust Confidence Limits 0.580–0.826; 95% Profile Likelihood Confidence Limits 0.603–0.791). These results are consistent with the mean survival times for each group as well as the observed survival curve, confirming the longer survival among Hispanic Blacks and shorter survival among non-Hispanic Blacks.

## 3. Methods of Goodness of Fit

Akaike Information Criterion (AIC), Deviance Information Criterion (DIC), and Bayesian Information Criterion (BIC) are the most commonly used models to measure the goodness of fit. DIC, a Bayesian measure of fit, is used for comparison of different models, for example, the use of public data by Congdon [31, 32]. The values of DIC can be either positive or negative. Models with lower values are considered better than others. DIC is similar to AIC and provides the same results as AIC when models with only fixed effects are fitted. BIC is an asymptotic result which assumes that the data distribution is an exponential family and can only be used to compare estimated models when numerical values of the dependent variable are identical for all estimates being compared. The BIC penalizes free parameters more than AIC. As is the case with AIC, given any two estimated models, the model with lower value of BIC is preferred.

### 3.1. The Log-Likelihood Function and Reparameterization

A reparameterization method from the Birnbaum-Saunders lifetime model was proposed by Ahmed et al. [33]. Later, Achcar et al. [34] considered a reparameterization from certain skewed models. A reparameterization method may be applied in terms of the log-likelihood functions considering data **x** = (*x*_1_, *x*_2_,…, *x*_*n*_) from the models described earlier which are given in the following.

The log-likelihood function from the EE model is given by

(5) ℓ(α,λ ∣ x)=nlog⁡(α)+nlog⁡(λ)+(α−1) ×∑i=1nlog⁡(1−exp⁡{−(λxi)})−λ∑i=1nxi.

Assume *ρ*_1_ = log⁡(*α*) and *ρ*_2_ = log⁡(*λ*). It is assumed that *ρ*_1_ and *ρ*_2_ are independently distributed. To obtain noninformative prior for *ρ*_1_ and *ρ*_2_, let a uniform prior distribution for *ρ*_*i*_ be *U*(−*c*_*i*_, *c*_*i*_), for all *i* = 1,2. Then the joint posterior density is given by

(6) p(ρ1,ρ2 ∣ x) =p(ρ1,ρ2)×exp⁡{nρ1+nρ2−nx−exp⁡{(ρ2)}+(exp⁡{(ρ1)}−1)×∑i=1nlog⁡(1−exp⁡{−xiexp⁡{(ρ2)}})}.

The log-likelihood function from the beta generalized exponentiated model is given by

(7) ℓ(α,λ,a,b ∣ x)=nlog⁡(α,λB(a,b))−λ∑i=1nxi+(aα−1) ×∑i=1nlog⁡(1−exp⁡{−(λxi)})+(b−1) ×∑i=1nlog⁡(1−[1−exp⁡{−(λxi)}]α).

Assume *ρ*_1_ = log⁡(*a*); *ρ*_2_ = log⁡(*b*); *ρ*_3_ = log⁡(*α*); and *ρ*_4_ = log⁡(*λ*). We further assume that *ρ*_1_, *ρ*_2_, *ρ*_3_, and *ρ*_4_ are independently distributed. To obtain noninformative prior for *ρ*_1_, *ρ*_2_, *ρ*_3_, and *ρ*_4_, let a uniform prior distribution for *ρ*_*j*_ be *U*(−*d*_*j*_, *d*_*j*_), for all *j* = 1,2, 3,4. Then the joint posterior density is given by

(8) p(ρ1,ρ2,ρ3,ρ4 ∣ x) =p(ρ1,ρ2,ρ3,ρ4)  ×[nlog⁡(eρ3+ρ4B(eρ1,eρ2))−eρ4∑i=1nxi+(eρ1+ρ3−1)    ×∑i=1nlog⁡(1−exp⁡{−eρ4xi})(eρ2−1)    ×(∑i=1nlog⁡(1−(1−exp⁡{−eρ4xi})eρ3))].

The log-likelihood function from the EW model is derived by

(9) ℓ(α,β,λ ∣ x) =nlog⁡(α)+nlog⁡(β)+nlog⁡(λ)+(α−1)  ×∑i=1nlog⁡(1−exp⁡{−(λxiβ)})  −λ∑i=1nxiβ+(β−1)∑i=1nlog⁡⁡(xi).

Assume *ρ*_1_ = log⁡(*α*); *ρ*_2_ = log⁡(*β*); and *ρ*_3_ = log⁡(*λ*). It is further assumed that *ρ*_1_, *ρ*_2_, and *ρ*_3_ are independently distributed. To obtain non-informative prior for *ρ*_1_, *ρ*_2_, and *ρ*_3_, let a uniform prior distribution for *ρ*_*k*_ be *U*(−*e*_*k*_, *e*_*k*_), for all *k* = 1,2, 3.

Then the joint posterior density is derived by

(10) p(ρ1,ρ2,ρ3 ∣ x)=p(ρ1,ρ2,ρ3)×exp⁡{n(ρ1+ρ2+ρ3)−eρ3∑i=1nxieρ2+(eρ1−1) ×∑i=1nlog⁡(1−exp⁡{−eρ3xieρ2}) +(eρ2−1)∑i=1nlog⁡(xi)}.

The log-likelihood function from the BIW model is given by

(11) ℓ(β,a,b ∣ x) =nlog⁡(βB(a,b))−a∑i=1nxi−β+(β−1)  ×∑i=1nlog⁡(xi)+(b−1)∑i=1nlog⁡(1−exp⁡{−xi−β}).

Assume *ρ*_1_ = log⁡(*β*); *ρ*_2_ = log⁡(*a*); and *ρ*_3_ = log⁡(*b*). We further assume that *ρ*_1_, *ρ*_2_, and *ρ*_3_ are independently distributed. To obtain non-informative prior for *ρ*_1_, *ρ*_2_, and *ρ*_3_, let a uniform prior distribution for *ρ*_*h*_ be *U*(−*f*_*h*_, *f*_*h*_), for all *h* = 1,2, 3.

Then the joint posterior density is given by

(12) p(ρ1,ρ2,ρ3 ∣ x) =p(ρ1,ρ2,ρ3)[nlog⁡(eρ1B(eρ2,eρ3))−eρ2∑i=1nxi−eρ1+(eρ1−1)×∑i=1nlog⁡(xi)+(eρ3−1)×∑i=1nlog⁡(1−exp⁡{−e−xi−eρ1})].

**Figure 3:**
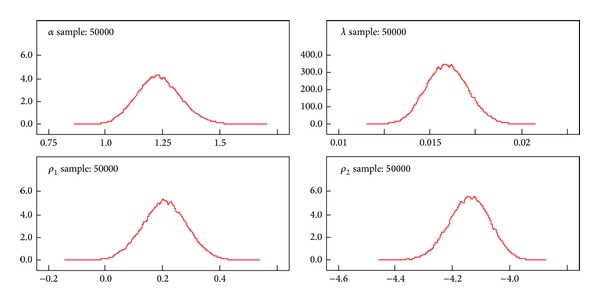
Kernel density of the posterior parameters in the case of exponentiated exponential for Black Hispanic females breast cancer patients (*n* = 298).

**Figure 4:**
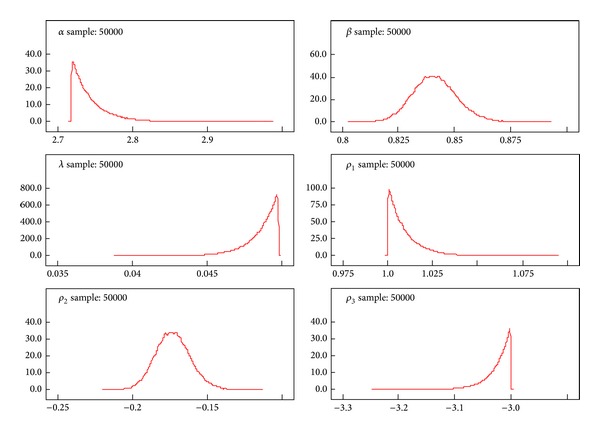
Kernel density of the posterior parameters for the exponentiated Weibull for Black Hispanic females breast cancer patients (*n* = 298).

**Figure 5:**
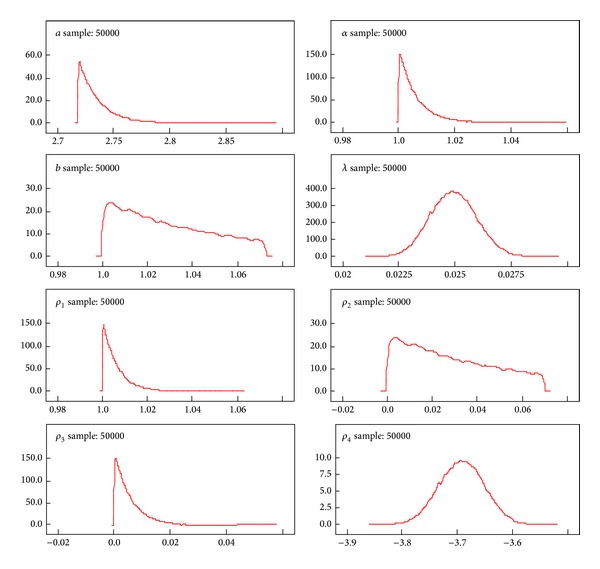
Kernel density of the posterior parameters in the case of beta generalized exponential for Black Hispanic females breast cancer patients (*n* = 298).

**Figure 6:**
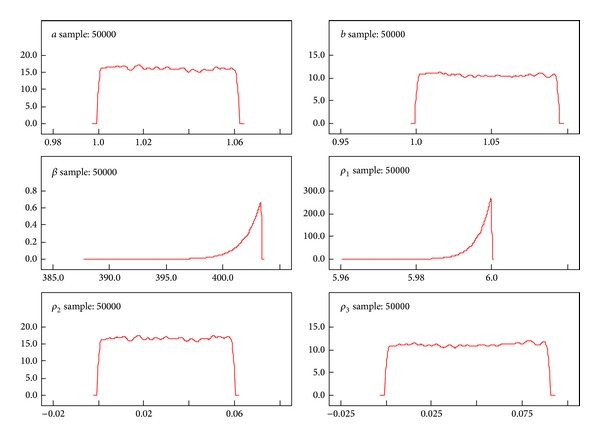
Kernel density of the posterior parameters in the case of BIW for Black Hispanic females breast cancer patients (*n* = 298).

A better performance of the posterior distributions for the parameters can be achieved with the reparameterization method.  Table 3 gives the results of the measures of goodness of fit for Black Hispanic females. Tables 4–7 summarize the results of the posterior parameters. Figures 3–6 show the posterior kernel densities for the parameters.

### 3.2. The Results of Goodness of Fit Tests and Posterior Inference for the Parameters from the Black Hispanic Survival Data

Table 3 includes the AIC, BIC, and DIC values for the EE, EW, BGE, and BIW models. Better model fit is inferred if the values of AIC, BIC, and DIC are the least. The data fits EE model better than the other models. The estimated value of AIC is the lowest (3136.72), while the DIC value is very close to AIC. Comparing the estimated values of all AIC, BIC, and DIC for the models, the EEM fits better for the survival days because it produces smaller values for all three criteria AIC, BIC, and DIC.

Table 4 summarizes the results of the posterior distribution of the parameters from the EE for the Black Hispanic breast cancer patients' survival data. In the Bayesian approach, the knowledge of the distribution of the parameters is updated through the use of observed data, resulting in what is known as the posterior distribution of the parameters. In the case of breast cancer data, we are interested in estimating the posterior distribution of the parameters assuming that observed random sample form an appropriate statistical probability distribution.

The values of the *ρ*_1_ and *ρ*_2_ are generated from the data and the results of the posterior distribution parameters *α* and *λ* are estimated using the MCMC method. Samples from a probability distribution can be generated using Markov Chain Monte Carlo which is a class of algorithms used in statistics [35]. The EE model is used to derive the log-likelihood function and the parameter values are assigned to the appropriate theoretical probability distributions. The summary results (mean, SD, MC error, median, and confidence intervals) of the parameters are obtained by using the WinBUGS software. In this process the early iterations up to 1,000 are ignored in order to remove any biases of estimated values of the parameters resulting from the value of **x** utilized to initialize the chain. This process is known as burn-in. The remaining samples are treated as if the samples are from the original distribution (after elimination of the burn-in samples). Fifty thousand (50,000) Monte Carlo repetitions were used to produce the inference for the posterior parameters as shown in Table 4. The graphical representation of the parameters' behavior is displayed in Figure 3. After 50,000 Monte Carlo repetitions, the kernel densities for both shape and scale parameters follow approximately symmetric distributions.

Table 5 shows the summary results of the posterior distribution of the parameters from the exponentiated Weibull. The Black Hispanic female breast cancer patients' survival data has been used for these results. The values *ρ*_1_, *ρ*_2_, and *ρ*_3_ have been generated from the data. Using the MCMC method, the results of the posterior distribution parameters *α*, *β*, and *λ* are estimated by setting the generated values. The log-likelihood function is derived from the EW model. Subsequently, the parameter values which are assigned to appropriate probability distributions are derived. The summary results (mean, SD, MC Error, median, and confidence intervals) of the parameters are derived using the WinBugs software. The graphical representation of distributions of the parameter behaviors has been summarized in Figure 4. The shape parameters *β* and  *ρ*_2_  show a normal distribution, while other model parameters show skewed distributions.

Table 6 shows the summary results of posterior distribution of the parameters from the BGE model. Black Hispanic female breast cancer patients' data has been used for these results. We used the WinBugs software to obtain the summary results (mean, SD, MC error, median, and confidence intervals) of the parameters. The graphical representation of the parameters for female in the case of BGE has been displayed in Figure 5. A symmetrical pattern of distribution is shown by the parameters *λ* and *ρ*_4_, while a nonsymmetrical distribution is shown by other parameters.

Table 7 shows the summary results of the posterior distribution of the parameters from the BIW model. The Black Hispanic female breast cancer patients' survival data has been used. The summary results which include (mean, SD, MC error, median, and confidence intervals) of the parameters have been derived by using the WinBugs software. The graphical representations of the parameters for females in the case of BIW model have been displayed in Figure 6. It is noted that the skewed distribution pattern is shown by parameters *β* and  *ρ*_1_ from the BIW, while other parameters show approximately uniform distributions.

## 4. The Bayesian Predictive Survival Model

Due to the current economic crisis, health care costs are increasing tremendously. It is important for health care researchers and providers to promptly identify the high risk population variables for several diseases. The goal is to identify and provide preventive interventions without significantly increasing the cost of management. Currently, predictive modeling is a popular technique used for high-risk assessment at very low costs. Health care providers and researchers will greatly benefit from predictive modeling both to improve present health care services and reduce future health care costs.

Predictive modeling is a process that can be applied to available healthcare data, for instance, identification of people who have high medical need and who are “at risk” for above-average future medical service utilization. We are deriving a novel Bayesian method which can predict the breast cancer survival days based on past data collected from patients.

The Bayesian predictive method is growing extremely popular, finding newer applications in the fields of health sciences, engineering, environmental sciences, business and economics, and social sciences, among others. The Bayesian predictive approach is used for the design and analysis of survival research studies in the health sciences. It is widely used to reduce healthcare costs and to economically allocate healthcare resources.

In this section, a predictive survival model for breast cancer patients is developed by using a novel Bayesian method. It is found that the Black Hispanic female breast cancer patients' data follow the EE model.

Let us assume that the data **x** = (*x*_1_,…, *x*_*n*_) represents *n* female breast cancer patients survival days that follow the EE model, and let *z* be a future response (or future survival days). The predictive density of *z* for the observed data **x** is

(13) p(z ∣ x)=∬p(z ∣ α,λ)p(α,λ ∣ x)dλ dα,

where *p*(*α*, *λ* | **x**) is the posterior density function and *p*(*z* | *α*, *λ*) represents the probability density function of a future response (*z*) that may be defined from model (1). The posterior density is given by

(14) p(α,λ ∣ x)=Ψ(x)L(α,λ ∣ x)p(α,λ),

where *L*(*α*, *λ* | **x**) is the likelihood function, *p*(*α*, *λ*) is the prior density for the parameters, and the reciprocal of the normalizing constant is

(15) $$\begin{array}{c} \Psi {\left(x\right)}^{-1}=\iint L\left(\alpha ,\lambda \;|\;x\right)p\left(\alpha ,\lambda \right)d\lambda \;d\alpha . \end{array}$$

To derive the likelihood function, let *x*_1_,…, *x*_*n*_ be a random sample of size *n* from model (1). Thus, **x** = (*x*_1_,…,*x*_*n*_)′ forms an observed sample. Then given a set of data **x** = (*x*_1_,…, *x*_*n*_) from (1), the likelihood function is given by

(16) L(α,λ ∣ x)∝(αλ)nexp⁡{−∑i=1n(λxi)} ×[∏i=1n(1−exp⁡{−(λxi)})α−1].

An estimation theory under uncertain prior information was discussed in detail by Ahmed [36]. Further details on Bayes and empirical Bayes estimates of survival and hazard functions of a class of distribution were discussed by Ahsanullah and Ahmed [21]. The estimation of lognormal mean by making use of uncertain prior information was also discussed by Ahmed and Tomkins [37]. The Bayesian predictive model from the Weibull life model, by means of a conjugate prior for the scale parameter and a uniform prior for the shape parameter, has been discussed at length by Khan et al. [25]. The prior density for the scale parameter (*λ*) can be given by

(17) p(λ)∝λexp⁡{−λ}, λ>0.

With reference from Khan et al. [25], the shape parameter, *α*, has a uniform prior over the interval (0, *α*), which is given as follows:

(18) $$\begin{array}{c} p\left(\alpha \right)\propto \frac{1}{\alpha },\;\alpha >0. \end{array}$$

Thus, the joint prior density is

(19) $$\begin{array}{c} p\left(\alpha ,\lambda \right)\propto \frac{\lambda \exp\left\{-\lambda \right\}}{\alpha },\;\alpha ,\lambda >0. \end{array}$$

Considering the prior density in (19), the posterior density of *α* and *λ* is given by

(20) p(α,λ ∣ x)=Ψ0(x)(α)n−1λn+1exp⁡{−∑i=1n(λxi)−λ} ×[∏i=1n(1−exp⁡{−(λxi)})α−1],

where Ψ_0_(**x**) is a normalizing constant.

### 4.1. Predictive Density for a Single Future Response

Let *z* be a single future response from the model specified by (1), where *z* is independent of the observed data. Then, the predictive density for a single future response (*z*) given **x** = (*x*_1_,…, *x*_*n*_) is

(21) p(z ∣ x)=∫α=0+∞∫λ=0+∞p(z ∣ α,λ)p(α,λ ∣ x)dλ dα,

where *p*(*z* | *α*, *λ*) may be defined from model (1). Thus, the predictive density for a single future response is given by

(22) p(z ∣ x)={Ψ1(x)∫α=0+∞∫λ=0+∞(α)nλ(n+2)×exp⁡{−(λz)}(1−exp⁡{−(λz)})α−1×exp⁡{−∑i=1n(λxi)−λ}×[∏i=1n(1−exp⁡{−(λxi)})α−1]dλ dα,for  z>0;  α,λ>0,0elsewhere,

where Ψ_1_(**x**) is a normalizing constant.

**Figure 7:**
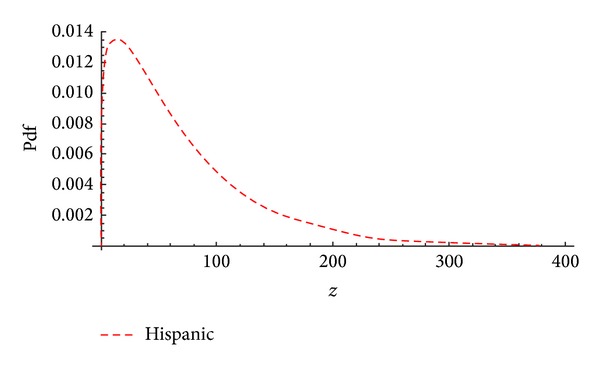
The predictive density for a single future response for Black Hispanic survival data.

Figure 7 shows the graphical representation of the predictive density based on the Black Hispanic female breast cancer patients' survival days. It is noted that the predictive density formed right skewed model.

The summary results of Black Hispanic female predictive means, standard errors, and predictive intervals for future survival days are given in Table 8. The predictive shape characteristics, raw moments, corrected moments, and measures of skewness and kurtosis are also presented in Table 8. These findings are very important for health care researchers to characterize future disease patterns and to make an effective future plans for prevention strategies for the diseases.

## 5. Results and Discussion

The mean ± SD of age at diagnosis is 54.11 ± 14.41 years for Black Hispanic group. The minimum age at diagnosis for Black Hispanic was 24 years. The mean ± SD of survival time (months) for Black Hispanic females was 71.38 ± 61.33. The majority of these patients were married.

The EE model is shown to be a better fit compared to other models for Black Hispanic survival data. The lowest DIC value for Black Hispanics is 3136.732. In the case of the EE model, mean ± SD for *α* and *λ* values is 1.234 ± 0.09684 and 0.01595 ± 0.001142, respectively. Rho (*ρ*) values are as follows: *ρ*_1_ = (−10,10) and *ρ*_2_ = (−10,10).

We used the Bayesian method to determine the inference for posterior parameters given the breast cancer survival model. Tables 4–7 summarize the inferences for the posterior parameters using less Markov Chain errors for Black Hispanic females. Figures 3–6, report the dynamic kernel densities for each of the parameters for Black Hispanic females. This helps us to observe the shapes of the kernel densities.

The graphical representation of Black Hispanic females' based on future survival times is shown in Figure 7. It should be noted that future survival times for Hispanic Black females show positively skewed distribution. Table 8 summarizes the predictive raw and corrected moments, predictive skewness and kurtosis, and predictive intervals for future response for Black Hispanic female future survival times.

## 6. Conclusions

There were four types of statistical probability models used to the Black Hispanic females cancer survival data. The exponentiated exponential model was found to be the best fitted model to the Black Hispanic females cancer survival data compared to the other widely used models.

The results of the predictive inference under the fitted model were obtained and it was noticed that the shape of the future survival model for Black Hispanic is positively skewed. Given the patient's current and past history of reported conditions, these models help the healthcare providers and researchers to predict a patient's future survival outcomes. Thus a combination of current knowledge and future predictions can be used to enhance and improve the rationales for better utilization of current facilities and planned allocation of future resources.

Descriptive statistics were obtained by using the SPSS software version 19.0. The geographic maps of the randomly selected nine states out of the twelve states were derived using the “Google fusion table” [38]. We used the SPSS version 19.0 software [39] to obtain basic summary statistics for the breast cancer survival times for Black Hispanic subset. To show the graphical representations of the predictive density for a single future response for Black Hispanic women, we used advanced computational software package called “Mathematica version 8.0” [40]. We used the same software to derive additional predictive inferences for the ethnicity about their survival times. We used the WinBugs software to check the goodness of fit tests, to derive the summary results of the posterior parameters, to determine the kernel densities of the parameters, and to carry out all related calculations.

## Figures and Tables

**Table 1:** Frequency distribution of the selected Black Hispanic breast cancer patients from the nine states.

States	Black Hispanic	
	Count	(%)
Georgia	94	31.5
Hawaii	1	0.3
Iowa	3	1.0
Michigan	44	14.8
New Mexico	5	1.7
Utah	4	1.3
Washington	9	3.0
California	67	22.5
Connecticut	71	23.8

Total	298	100

**Table 2:** Given below is the marital status, survival time, and age at diagnosis for female Black Hispanic breast cancer patients. Age at diagnosis and survival time are classified by mean, standard deviation (SD), median, range, and quartiles. Marital status is classified into six categories and their respective frequencies are reported.

Characteristics	Categories	Black Hispanic
Age at diagnosis (years)	Mean	54.11
	SD	14.41
	Median	52
	Quartile	
	*Q*_1_	44
	*Q*_2_	52
	*Q*_3_	64
	Variance	207.65

Survival time (months)	Mean	71.38
	SD	61.33
	Median	50.50
	Quartile	
	*Q*_1_	25
	*Q*_2_	50.50
	*Q*_3_	101.25
	Variance	3761.04

Marital status at diagnosis	Single	57
	Married	124
	Separated	7
	Divorced	51
	Widowed	50
	Unknown	9

**Table 3:** Selection of the best model for Black Hispanic females on the basis of AIC, BIC, and DIC criterions.

Model criterions	AIC	BIC	DIC
Exponentiated exponential	3136.72	3144.11	3136.732
Exponentiated Weibull	3184.25	3191.64	3182.328
Beta generalized exponential	3262.20	3276.98	3256.208
Beta inverse Weibull	3323.65	3334.74	3317.650

**Table 4:** Summary of the results of the posterior parameters from exponentiated exponential for Black Hispanic females breast cancer patients (*n* = 298).

Node	Mean	SD	MC error	Median	95% CI	Sample
*α*	1.234	0.09684	8.94*E* − 04	1.231	(1.053, 1.434)	50000
*λ*	0.01595	0.001142	1.06*E* − 05	0.01593	(0.01377, 0.01824)	50000
*ρ*_1_	0.2073	0.07844	7.26*E* − 04	0.2076	(0.05193, 0.3608)	50000
*ρ*_2_	−4.141	0.07182	6.69*E* − 04	−4.139	(−4.285, −4.004)	50000

	Dbar		Dhat		pD	

Zeros	3134.730		3132.720		2.006	
Total	3134.730		3132.720		2.006	

Dbar: post.mean of −2log⁡*L*; Dhat: −2log⁡*L* at post.mean of stochastic nodes.

*ρ*_1_~dunif (−10,10); *ρ*_2_~dunif (−10,10).

**Table 5:** Summary results of the posterior parameters for exponentiated Weibull for Black Hispanic females breast cancer patients (*n* = 298).

Node	Mean	SD	MC error	Median	95% CI	Sample
*α*	2.743	0.02486	2.43*E* − 04	2.736	(2.719, 2.809)	50000
*β*	0.8414	0.01005	9.04*E* − 05	0.841	(0.8227, 0.8625)	50000
*λ*	0.04858	0.001167	1.31*E* − 05	0.04892	(0.04547, 0.04976)	50000
*ρ*_1_	1.009	0.008985	8.77*E* − 05	1.006	(1.000, 1.033)	50000
*ρ*_2_	−0.1728	0.01193	1.07*E* − 04	−0.1731	(−0.1952, −0.1479)	50000
*ρ*_3_	−3.025	0.02458	2.77*E* − 04	−3.018	(−3.091, −3.001)	50000

	Dbar		Dhat		pD	

Zeros	3181.290		3180.250		1.039	
Total	3181.290		3180.250		1.039	

Dbar: post.mean of −2log⁡*L*; Dhat: −2log⁡*L* at post.mean of stochastic nodes.

*ρ*_1_~dunif (1, 2); *ρ*_2_~dunif (−10, 1); *ρ*_3_~dunif (−4, − 3).

**Table 6:** Summary results of the posterior parameters in the case of BGE for Black Hispanic females breast cancer patients (*n* = 298).

Node	Mean	SD	MC error	Median	95% CI	Sample
*a *	2.735	0.01647	1.64*E* − 04	2.73	(2.719, 2.779)	50000
*α*	1.006	0.005935	5.38*E* − 05	1.004	(1.000, 1.022)	50000
*b *	1.029	0.02016	1.55*E* − 04	1.025	(1.001, 1.069)	50000
*λ*	0.02494	0.001025	7.24*E* − 06	0.02492	(0.02298, 0.02698)	50000
*ρ*_1_	1.006	0.005986	5.94*E* − 05	1.004	(1.00, 1.022)	50000
*ρ*_2_	0.02805	0.01952	1.51*E* − 04	0.02482	(0.001068, 0.06674)	50000
*ρ*_3_	0.005888	0.005865	5.32*E* − 05	0.004063	(1.48*E* − 04, 0.02165)	50000
*ρ*_4_	−3.692	0.04112	2.91*E* − 04	−3.692	(−3.773, −3.613)	50000

	Dbar		Dhat		pD	

Zeros	3255.200		3254.200		1.004	
Total	3255.200		3254.200		1.004	

Dbar: post.mean of −2log⁡*L*; Dhat: −2log⁡*L* at post.mean of stochastic nodes.

*ρ*_1_~dunif (1,2), *ρ*_2_~dunif (0,0.07), *ρ*_3_~dunif (0,1), and *ρ*_4_~dunif (−4, −3).

**Table 7:** Summary of the posterior parameters in the case of BIW for Black Hispanic females breast cancer patients (*n* = 298).

Node	Mean	SD	MC error	Median	95% CI	Sample
*a *	1.031	0.01789	8.38*E* − 05	1.03	(1.001, 1.06)	50000
*b *	1.047	0.02736	1.21*E* − 04	1.047	(1.002, 1.092)	50000
*β*	402.1	1.344	0.01014	402.5	(398.4, 403.4)	50000
*ρ*_1_	5.997	0.003353	2.53*E* − 05	5.998	(5.988, 6.000)	50000
*ρ*_2_	0.03002	0.01736	8.14*E* − 05	0.02997	(0.001484, 0.05849)	50000
*ρ*_3_	0.0454	0.02614	1.16*E* − 04	0.04559	(0.00228, 0.08786)	50000

	Dbar		Dhat		pD	

Zeros	3317.650		3317.650		−0.000	
Total	3317.650		3317.650		−0.000	

Dbar: post.mean of −2log⁡*L*; Dhat: −2log⁡*L* at post.mean of stochastic nodes.

*ρ*_1_~dunif (0,6), *ρ*_2_~dunif (0,0.06), and *ρ*_3_~dunif (0,0.09).

**Table 8:** Predictive inference for Black Hispanic female breast cancer patients survival data.

Summary	Black Hispanic
Mean	89.7881
SE	1.2802
Raw moments	
*m*_1_	89.7881
*m*_2_	11339.90
*m*_3_	2.0034 × 10^6^
*m*_4_	4.39016 × 10^8^
Corrected moments	
*μ*_1_	89.7881
*μ*_2_	3277.84
*μ*_3_	396582
*μ*_4_	7.3029 × 10^7^
Skewness and Kurtosis	
*β*_1_	4.46582
*β*_2_	6.79703
*γ*_1_	2.11325
*γ*_2_	3.79703
Predictive intervals	
90%	(28.532, 325.350)
95%	(24.120, 332.450)
98%	(22.013, 346.092)
99%	(21.051, 358.301)
